# Risk of accidents and unintentional injuries in men and women with attention deficit hyperactivity disorder across the adult lifespan

**DOI:** 10.1111/acps.13524

**Published:** 2022-12-14

**Authors:** Berit Libutzki, Benno Neukirch, Sarah Kittel‐Schneider, Andreas Reif, Catharina A. Hartman

**Affiliations:** ^1^ Department of Psychiatry, Interdisciplinary Center Psychopathology and Emotion regulation (ICPE) University of Groningen, University Medical Center Groningen Groningen The Netherlands; ^2^ Hochschule Niederrhein University of Applied Sciences Krefeld Germany; ^3^ Department of Psychiatry, Psychotherapy and Psychosomatic Medicine University Hospital of Würzburg Würzburg Germany; ^4^ Department of Psychiatry, Psychosomatic Medicine and Psychotherapy University Hospital Frankfurt Frankfurt am Main Germany

**Keywords:** accidental injuries, attention deficit disorder with hyperactivity (ADHD), comorbidity, real‐world evidence, wounds and injuries

## Abstract

**Introduction:**

Attention deficit hyperactivity disorder (ADHD) is associated with risk‐taking behavior, leading to accidents and unintentional injuries (summarized here as incidents). Main aim of this study is to determine if men and women with and without ADHD differ in the risk of mild (treated outpatient) and severe (treated inpatient) incidents across the adult lifespan (age groups: 18–29; 30–59, and ≥60 years). Secondary aim: investigate the role of comorbid mental disorders and drugs for the treatment of these comorbidities, and ADHD‐medication.

**Methods:**

Using anonymized German claims data (*N* = 4,575,027), adults with ADHD diagnosis during 2016–2019 (*N* = 17,041) were compared with a 1:4 age and sex‐matched group without ADHD diagnosis. Regression analyses statistically tested group differences.

**Results:**

Incidents occur in a U‐shaped form across the adult lifespan. Individuals with ADHD show the same pattern but at a substantially increased risk of both mild and severe incidents throughout the lifespan. Women without ADHD are at lower risk in young adulthood than men but at higher risk in older adulthood. Women with ADHD show the same pattern for severe incidents, but for mild incidents they have the highest risk throughout the lifespan. Co‐occurring anxiety disorder and the use of psycholeptics and ADHD‐medication decreased the incident risk.

**Conclusion:**

We extend available knowledge which has hitherto focused on young adult males and traffic accidents. ADHD is associated with increased incidents across the adult lifespan, with distinct patterns regarding age, sex, and incident severity. An accurate diagnosis of ADHD in adulthood provides the first step towards prevention of accidents and unintentional injuries.

**Significant Outcomes**

- Throughout their entire adult lifespan, adults with attention deficit hyperactivity disorder (ADHD) are at a significantly higher risk for accidents and unintentional injuries (summarized as incidents) as compared with adults without ADHD.
- Especially women with ADHD are at an increased risk for incidents, already as young adults.
- ADHD‐medication, comorbid anxiety disorder and psycholeptics decrease the risk for incidents in adults with ADHD. Hence, early diagnosis and treatment may reduce incident risk.

**Limitations**

- Individuals with ADHD were identified within the claims dataset based on an ICD‐10 diagnosis from the inpatient and outpatient setting, including primary care. Therefore, the study sample built upon the administrative prevalence of diagnosed individuals may represent more severe cases compared with individuals with ADHD in in the general population, which may have led to an overestimation of the incident risk associated with ADHD.
- The study includes adults with ADHD across the entire adult lifespan and stratifies within age groups. Nevertheless, the observed time was restricted to a one‐year observation which may have led to an underestimation of the incident risk associated with ADHD across the adult lifespan.
- Individuals with ADHD in this study may have received ADHD‐medication before or after an incident (sequence of events was not tracked). Hence, the observed protective overall effect of ADHD‐medication against incidents may be underestimated.

## 1 INTRODUCTION

Attention deficit hyperactivity disorder (ADHD) in adults has been increasingly recognized as an impairing mental health condition.^1^ Part of this impairment involves a higher risk of having accidents.^2^, ^3^ It is also known that injuries are a key driver of inpatient care costs for children with ADHD.^4^ Compared with childhood, research on ADHD in adulthood is strongly lagging behind.^5^ Consequently, our knowledge on ADHD in adulthood is still highly fragmented and this also holds for the occurrence of accidents. Traffic accidents have been studied in young adults with ADHD,^6^, ^7^ especially in males, but we know much less about (other) accidents and unintentional injuries in women and in older age groups. While potentially less severe than traffic accidents, other accidents and unintentional injuries may be more frequent and as such importantly contributing to the burden of adult ADHD. In addition, their incidence may differ in males and females and in different age groups. Collectively we will below refer to all types of accidents and unintentional injuries as incidents. In the general population, it has been reported that incidents tend to decrease and stabilize with age until old age, when they start to increase again (which can be plotted as a U‐shaped incidence curve).^8^, ^9^ This may be the case for individuals with ADHD as well, but again, literature on the distribution of incidents across the adult lifespan is very scarce.^3^ Moreover, common comorbid conditions of ADHD and (co‐) medication may play a role in our understanding of incidents, as they may potentially reduce (e.g., anxiety disorders [AD])^10^ or enhance (e.g., substance use disorders [SUDs]) the risk of incidents.^2^ The aim of the present study was therefore to determine the occurrence of incidents in adults diagnosed with ADHD compared with adults without ADHD. Differentiating between severe (treated inpatient) and mild (treated outpatient) incidents, we studied if the occurrence of incidents changes with age and sex. As a secondary aim we investigate the role of comorbid mental disorders and medication in adults with ADHD compared with those without ADHD.

## 2 MATERIALS AND METHODS

### 2.1 Sample

Approximately 90% of the overall German population is insured in statutory health insurances (SHI). For this study, claims data from the Institute for Applied Health Research Berlin (InGef) database was used. This database contains approximately 5 million member‐records and is representative of the German population in terms of age and sex and shows good overall accordance in morbidity, mortality, and drug usage.^11^ The study sample consists of adults diagnosed with ADHD at least once in the years 2016–2019 as documented by a F90 ICD‐10 GM diagnosis (International Statistical Classification of Diseases and Related Health Problems, 10th revision, German modification: F90.0, F90.1, F90.8, F90.9) as a primary, secondary, or tertiary care confirmed outpatient or inpatient (main or secondary) diagnosis.

For each individual identified with ADHD, four adults identical in age and sex but without ADHD diagnosis were randomly drawn. Index was set as at the quarter of first ADHD diagnosis at which individuals must have been at least 18 years old. For individuals without ADHD, a pseudo‐index at the same day as their matched ADHD comparator was set. After index, each individual was observed for 1 year for the occurrence of accidents and unintentional injuries, as well as medication, including drugs for the treatment of (ADHD‐medication), psycholeptics, antidepressants, drugs for the treatment of addictive disorders and the occurrence of pre‐defined comorbidities (AD, depressive disorder [DD], SUD); coding, see Table S1. During this time, individuals must have had continuous insurance coverage. We differentiated between mild and severe incidents based on the origin of diagnoses. Inpatient primary diagnoses, indicating the cause of hospitalization as per German coding guidelines,^12^ were counted as ‘severe’ and inpatient secondary diagnoses or outpatient secured diagnoses were counted as ‘mild’. In case individuals had multiple incidents, the more severe was relevant for subgroup assignment.

### 2.2 Ethics approval and patient consent statement

All data are anonymized in the research database to comply with German data protection regulations. Institutional review board/ethical approval and informed consent was not required.

### 2.3 Statistical analyses

Demographic information, occurrence of accidents and unintentional injuries and mortality rates are described stratified by sex and age (“young adults” 18–29 years, “adults” 30–59 years, “older adults” ≥60 years) groups. To formally test if there are age and sex specific patterns in mild and severe incidents that differ among individuals with and without ADHD, we conducted multinomial logistic regression analyses. In all regression models, age was analyzed in categories to accommodate for the presence of potential non‐linear effects across the lifespan. Specifically, we studied if ADHD, sex and age and the interactions of ADHD with sex and age (sex by ADHD, age by ADHD; sex by age, sex by age by ADHD) were associated with the likelihood of incidents according to incident severity.

In addition, we tested in a second model (by adding to the first regression model), effects of the presence of (comorbid) mental disorders and medication use and the respective two‐way interactions with ADHD (ADHD by DD, ADHD by AD, ADHD by SUD, as well as ADHD by drugs for the treatment of addictive disorders, psycholeptics and antidepressants). This informed us on whether findings of our main analysis were altered by taking account of comorbidities and co‐medication in adults with ADHD. In a third model, testing if ADHD‐medication altered the occurrence of incidents, we included only individuals diagnosed with ADHD (ADHD‐medication is very rarely prescribed in individuals without ADHD). Main effects were ADHD‐medication, sex and age as well as two‐way interaction effects (sex by ADHD‐medication, age by ADHD‐medication) and the three‐way interaction effect sex by age by ADHD‐medication.

Estimates from the multinomial regression analyses were shown as risk ratios (RR) with 95% confidence intervals (CI) and significance tested at a level of *p* < 0.05 using *z* tests. For all analyses, the statistical software Microsoft R Open 3.5.0 was used.

## 3 RESULTS

### 3.1 Sample retrieval and descriptive findings

Out of 4,575,027 individuals insured in the database from January 2016 to December 2019, 70,758 individuals with diagnosed ADHD were identified. Among those, 17,041 were above 18 years old, continuously insured, and therefore included in the analyses.

**FIGURE 1:**
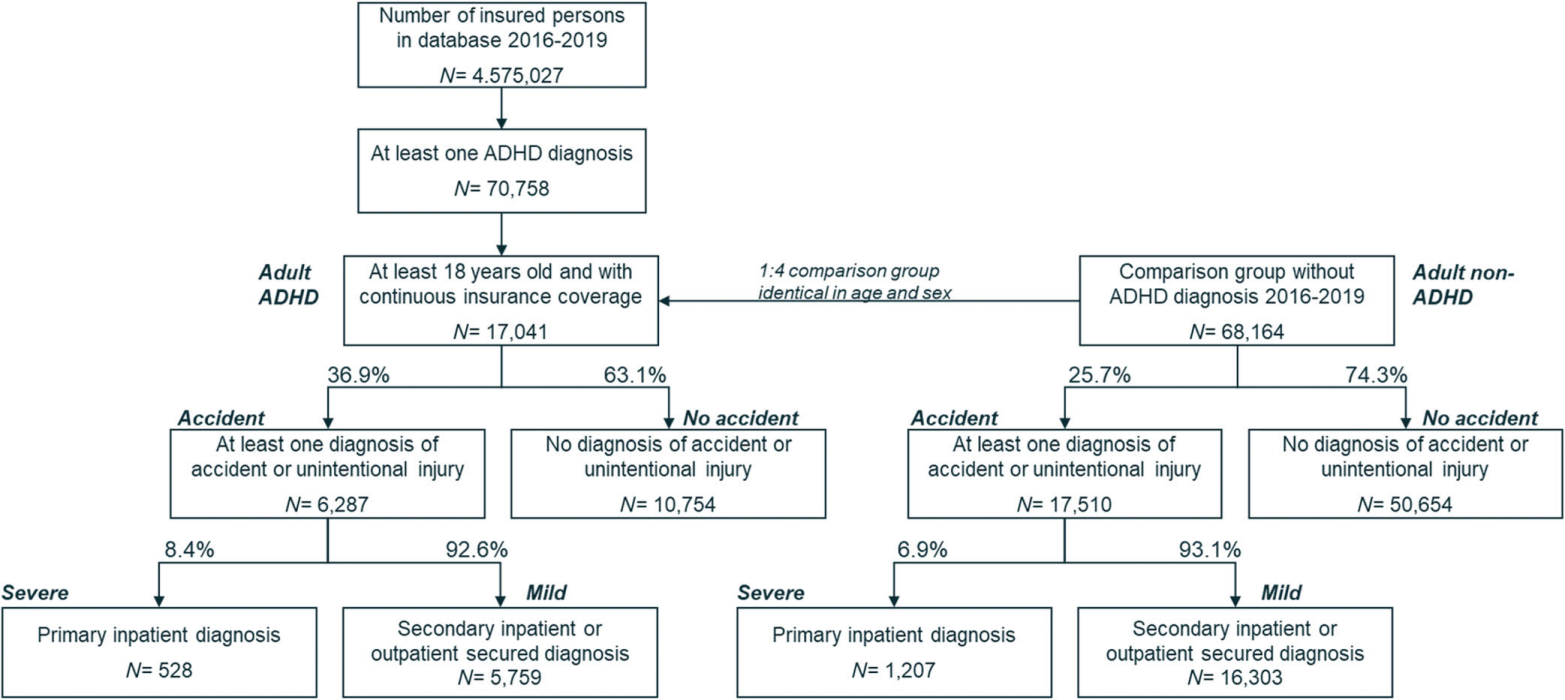
Flow‐chart of selection of adults diagnosed with ADHD and sex and age matched comparison group and occurrence of accidents and unintentional injuries. ‘Incident’ includes all occurrences either documented as accidents or unintentional injuries. ADHD, attention deficit hyperactivity disorder

Figure 1 shows (descriptive findings, not yet statistically tested) that the occurrence of incidents (mild and severe combined) is elevated in individuals with ADHD compared with those without ADHD (36.9% vs. 25.7%) and that the share of severe incidents relative to mild incidents is higher for individuals with ADHD compared with individuals without ADHD (8.4% vs. 6.9%).

Table 1 provides demographic information and shows that most adults diagnosed with ADHD were males (male‐to‐female ratio of approximately 2:1). Mean age was 32.46 years (±SD 15.49). Females with ADHD were older than males with ADHD (females 37.61 ± SD 17.73; males 29.86 ± SD 13.50). In line with this, the male‐to‐female ratio was approximately 1:3 in young adulthood, 5:7 in middle adulthood and 13:9 in older‐aged adults. Mortality was twice as high in individuals with ADHD compared with those without ADHD (0.6% vs. 0.3%) and was higher in females with ADHD than males with ADHD. The higher percentage of incidents in individuals with ADHD compared with individuals without ADHD was visible across the sex and age groups.

**TABLE 1:** Demographics, mortality, and occurrence of incidents stratified by sex and age

	ADHD			Non‐ADHD		
Sex at index^a^						
	Female	Male	Total	Female	Male	Total
*N* (%)	5711 (34)	11,330 (66)	17,041(100)	22,844 (34)	45,320 (66)	68,164 (100)
Age (years) at index of total *N* (%)^a^						
18–29 years	2499 (44)	7432 (66)	9931 (58)	9996 (44)	29,728 (66)	39,724 (58)
30–59 years	2558 (45)	3433 (30)	5991 (35)	10,232 (45)	13,732 (30)	23,964 (35)
≥60 years	654 (11)	465 (4)	1119 (7)	2616 (11)	1860 (4)	4476 (7)
Death in year of observation^b^ of total *N* (%)						
Total	43 (0.8)	59 (0.5)	102 (0.6)	108 (0.5)	106 (0.2)	214 (0.3)
18–29 years	<5	6 (0.1)	7 (0.1)	0 (0.0)	8 (0.0)	8 (0.0)
30–59 years	8 (0.3)	21 (0.6)	29 (0.5)	17 (0.2)	27 (0.2)	44 (0.2)
≥60 years	34 (5.2)	32 (6.9)	66 (5.9)	91 (3.5)	71 (3.8)	162 (3.6)
Percentage of individuals with at least one accident or unintentional injury out of all individuals with and without ADHD during the year of observation^b^						
Any accidents and unintentional injuries of total *N* (%)						
Total	2130 (37.3)	4157 (36.7)	6287 (36.9)	5723 (25.1)	11,787 (26.0)	17,510 (25.7)
18–29 years	965 (38.6)	2855 (38.4)	3820 (38.5)	2490 (24.9)	8372 (28.2)	10,862 (27.3)
30–59 years	881 (34.4)	1121 (32.7)	2002 (33.4)	2.352 (23.0)	2942 (21.4)	5294 (22.1)
≥60 years	284 (43.4)	181 (38.8)	465 (41.5)	881 (33.7)	473 (25.4)	1354 (30.3)
Severe accidents and unintentional injuries of total *N* (%)						
Total	180 (3.2)	348 (3.1)	528 (3.1)	356 (1.6)	851 (1.9)	1207 (1.8)
18–29 years	67 (2.7)	252 (3.4)	319 (3.2)	123 (1.2)	646 (2.2)	769 (1.9)
30–59 years	61 (2.4)	72 (2.1)	133 (2.2)	95 (0.9)	143 (1.0)	238 (1.0)
≥60 years	52 (8.0)	24 (5.2)	76 (6.8)	138 (5.3)	62 (3.3)	200 (4.5)
Mild accidents and unintentional injuries of total *N* (%)						
Total	1950 (34.1)	3809 (33.6)	5759 (33.8)	5367 (23.5)	10,936 (24.1)	16,303 (23.9)
18–29 years	898 (35.9)	2603 (35.0)	3.501 (35.3)	2367 (23.7)	7726 (26.0)	10,093 (25.4)
30–59 years	820 (32.1)	1049 (30.6)	1869 (31.2)	2257 (22.1)	2799 (20.4)	5056 (21.1)
≥60 years	232 (35.5)	157 (33.7)	389 (34.7)	743 (28.4)	411 (22.1)	1.154 (25.8)

Abbreviation: ADHD, attention deficit hyperactivity disorder.

^a^ Age and sex distribution of individuals without ADHD is identical to individuals with ADHD because of the the 1:4 matched comparison group.

^b^ Reasoning: mortality is measured during the one‐year period, not at index.

**FIGURE 2:**
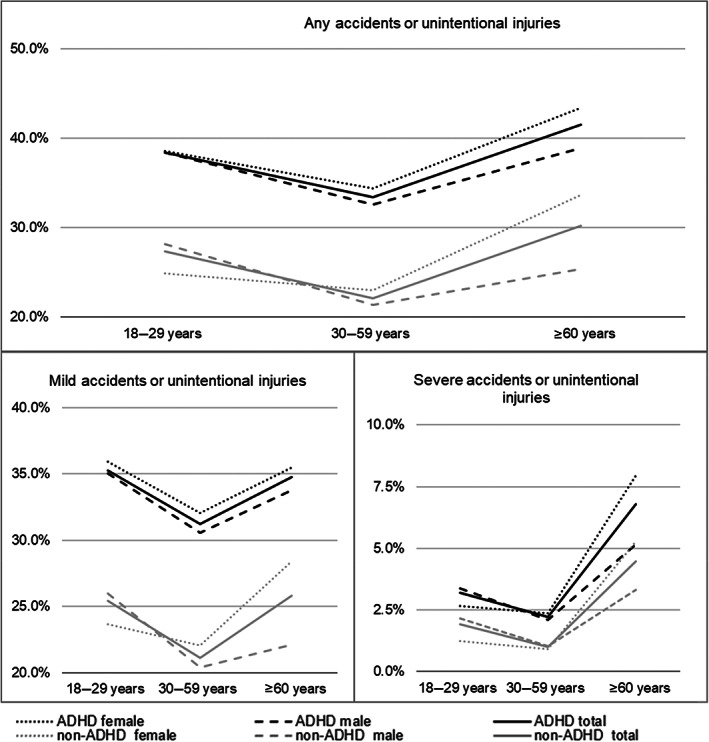
Occurrence of incidents across adulthood for males and females with and without ADHD, overall (top) and stratified by incident severity (bottom). Different scales are used for graphs; ‘Any incident’ depicts the sum of all ‘mild’ and ‘severe’ accidents and unintentional injuries. ADHD, attention deficit hyperactivity disorder

Figure 2 illustrates the percentages of accidents and unintentional injuries in men and women with and without ADHD in different phases of the adult lifespan. The top graph combines mild and severe incidents while the lower left and lower right graphs separately plot mild and severe incidents. Figure 2 reveals a U‐shaped form across the lifespan for both mild and severe incidents and for individuals with and without ADHD. It is shown that mild incidents form the vast majority of all incidents compared with severe incidents that are, by comparison, rarer. Thus, mild and severe incidents were analyzed separately in multinomial regression analyses.

Table S2 provides all percentages as shown in Figure 2. Table S3 provides a list of the types of incidents in individuals with and with ADHD.

### 3.2 Multinomial regression analyses

#### 3.2.1 Main analysis: ADHD, age, and sex in relation to incident occurrence

Table 2 shows the main findings using multinomial regression analyses, with ADHD (present/absent), sex and life phase and their interactions as predictors and mild and severe incidents, respectively, as outcomes.

**TABLE 2:** Multinomial logistic regression: main analysis assessing the role of ADHD, age, sex and interaction effects in relation to the likelihood of accidents and unintentional injuries

Term	Risk ratios (RR)	Standard error	*p* value	Lower (95%) confidence interval	Upper (95%) confidence interval
Mild accidents or unintentional injuries (reference category: no incidents)					
Intercept	0.36	0.01	<0.001	0.35	0.37
ADHD (1) versus non‐ADHD (0)	1.57	0.03	<0.001	1.49	1.66
Age group 30–59 years	0.72	0.03	<0.001	0.68	0.75
Age group 60+ years	0.82	0.06	0.001	0.73	0.92
Sex (female 1, male 0)	0.87	0.03	<0.001	0.83	0.92
ADHD/non‐ADHD: Sex	1.18	0.06	0.003	1.06	1.32
ADHD/non‐ADHD: age group 30–59 years	1.11	0.05	0.037	1.01	1.23
ADHD/non‐ADHD: age group 60+ years	1.19	0.12	0.145	0.94	1.49
Age group 30–59 years: sex	1.27	0.04	<0.001	1.17	1.38
Age group 60+ years: sex	1.66	0.08	<0.001	1.43	1.93
Age group 30–59 years: sex: ADHD/non‐ADHD	0.83	0.09	0.026	0.70	0.98
Age group 60+ years: sex: ADHD/non‐ADHD	0.67	0.16	0.010	0.49	0.91
Severe accidents or unintentional injuries (reference category: no incidents)					
Intercept	0.03	0.04	<0.001	0.03	0.03
ADHD (1) versus non‐ADHD (0)	1.82	0.08	<0.001	1.57	2.11
Age group 30–59 years	0.44	0.09	<0.001	0.37	0.53
Age group 60+ years	1.48	0.14	0.004	1.13	1.93
Sex (female 1, male 0)	0.54	0.10	<0.001	0.45	0.66
ADHD/non‐ADHD: sex	1.46	0.17	0.027	1.05	2.05
ADHD/non‐ADHD: age group 30–59 years	1.29	0.17	0.121	0.94	1.78
ADHD/Non‐ADHD: Age group 60+ years	1.04	0.26	0.889	0.62	1.73
Age group 30–59 years: sex	1.68	0.17	0.002	1.21	2.33
Age group 60+ years: sex	3.28	0.19	<0.001	2.28	4.73
Age group 30–59 years: sex: ADHD/non‐ADHD	0.88	0.28	0.636	0.51	1.52
Age group 60+ years: sex: ADHD/non‐ADHD	0.64	0.35	0.199	0.32	1.27

*Note*: Residual deviance: 111886.2; AIC: 111934.2; prediction accuracy 0.7207089.

Throughout the lifespan and regardless of sex, individuals with ADHD are at a significantly higher risk for both mild and severe incidents than individuals without ADHD (RR mild 1.57; severe 1.82; see also Figure 2).

Table 2 shows that, regardless of ADHD status, the risk of mild incidents is significantly lower in middle‐adulthood (RR 0.72) and older age (RR 0.82) compared with young adulthood. While the risk of severe incidents is likewise lower in middle adulthood (RR 0.44), it is higher in older adulthood (RR 1.48). Figure 2, right bottom compared with left bottom, illustrates, this steep increase in severe accidents in older age (compared with only a slight increase from mid‐ to older adulthood in mild incidents).

When differentiating across the different life phases, the age group by sex interactions in Table 2 show that, both in middle‐adulthood and older age, women are at a significantly higher risk than men to have mild (30–59/≥60 years: RR 1.27/1.66) and especially severe incidents (30–59/≥60 years: RR 1.68/3.28), see also Figure 2. This higher risk for women compared with men to have more incidents is most outspoken for women with ADHD, as indicated by the sex by ADHD interactions for mild (RR 1.18) and severe incidents (1.46), see Table 2. This excess risk found particularly for women with ADHD holds equally across the lifespan for severe accidents (i.e., no significant sex by ADHD by age‐group interaction). The significant sex by ADHD by age‐group interactions for mild incidents (middle aged and older aged adults 30–59/≥60 years: RR 0.83/0.67) indicate that the excess risk for women with ADHD compared with women without ADHD is strongest in young adulthood. Indeed, Figure 2, left bottom, shows that while women without ADHD have a clear reduced risk during young adulthood compared with men without ADHD, women with ADHD have a slightly higher risk compared with men with ADHD.

#### 3.2.2 Secondary analyses: additional role of comorbidity and medication

In a second regression model, we added the presence of mental disorders (AD, DD, and SUD) and medication (drugs for the treatment of addictive disorders, psycholeptics and antidepressants) to the initial regression model. For the percentage of individuals with and without ADHD with these mental disorders and medication intake see Table S4. Results of this secondary analysis indicated that the presence of AD in individuals with ADHD decreases the risk of mild incidents (RR 0.82) and that the intake psycholeptics in individuals with ADHD decreases the risk of severe incidents (RR 0.58) (Table S5). Adding both mental disorders (AD, DD, and SUD) as well as medication for these disorders to the model, the pattern of estimates for ADHD, age and sex as tested in the main analysis remained similar.

In a third regression model, within the ADHD group (Table S6), it was tested if the risk for incidents would be reduced if individuals with ADHD received ADHD‐medication, and if this would hold both in males and females and across the lifespan. For mild incidents, we find that the intake of ADHD‐medication decreases the risk significantly (RR 0.76) and that this is independent of sex and age (except for middle‐aged adults, who, compared with young adults, had a significantly higher risk for incidents even with medication intake). Within the notably smaller group of severe incidents (i.e., because of the reduced statistical power), no statistically significant effects of ADHD‐medication on occurrence of severe incidents are found, although the risk ratio estimate is similar (RR 0,76, *p* = 0.062).

## 4 DISCUSSION

This study shows that ADHD in adulthood is associated with an increased risk for incidents. While this was partly known from previous research, we substantially extend this knowledge in relation to age differences, sex differences, presence of comorbid conditions and medication. That is, while the existing literature has so far predominantly focused on traffic accidents that occur particularly in young adult males, we evaluated a much broader set of incidents and differentiated between mild and severe incidents. In addition, we studied men and women separately and differentiated into young, middle‐aged and older adults. Our findings showed, first, that across the lifespan the prevalence of incidents has a U‐shaped distribution in individuals with and without ADHD, with the lowest prevalence in middle adulthood. Importantly, the risk of incidences is higher in individuals with ADHD across the adult lifespan. Second, our findings showed important sex differences. For severe incidents, young adult men with and without ADHD have the highest risk compared with young adult women with and without ADHD. This reverses in older adulthood, where women with and without ADHD have the highest risk compared with their respective male counterparts. For mild incidents, this pattern holds for men and women without ADHD, but not for men and women with ADHD: women with ADHD are at highest risk for mild incidents across the adult lifespan. Third, we showed that for individuals with ADHD, the risk of incidents is decreased by comorbid AD (mild incidents) and intake of psycholeptics (severe incidents). In addition, use of ADHD‐medication is associated with a decreased risk of mild incidents especially in young and older age when incident risk is highest.

An important finding in this study is the U‐shaped occurrence of both mild and severe incidents. In literature, mostly driving behavior in relation to traffic accidents has been studied. A high risk of traffic accidents in young adulthood is generally explained by inexperience and higher risk‐taking behavior that characterizes young adults.^13^ Older adults, also show a particular high risk of incidents, mostly explained by household‐related incidents, such as falls.^3^, ^14^ The age‐related decline of general health, i.e. slower reflexes, sensory impairment and brittle bones, may be risk factors for incidents in older adults and they also may increase the frequency and severity of incidents.^15^ Our findings revealed that this U‐shape also holds for individuals with ADHD—yet, importantly, at an highly elevated level throughout the lifespan. This raises the question if the aforementioned mechanisms of high risk‐taking behavior in young adults and lower health status in older adults explaining incident proneness are more pronounced in individuals with ADHD. Indeed, the core symptoms of ADHD speak to this: impulsiveness, hyperactivity (or its adult variant: restlessness) and inattentiveness contribute to risk‐taking behavior,^16^, ^17^, ^18^ and this likely plays a role throughout the lifespan. Inattention might have an important role in young adulthood, especially in traffic accidents,^16^ where driving behavior is less automatized because of the lack of experience.^19^ With respect to mid‐to older adulthood, it has been shown that ADHD is associated with earlier than average loss of health, including earlier onset and increased occurrence of neurodegenerative conditions.^20^, ^21^ Thus, mid‐adults and older adults with ADHD are less able to compensate for age‐related decreases in sensory‐motor functioning. In addition, literature has indicated that individuals with ADHD show an elevated mortality rate compared with individuals without ADHD and that this is mainly driven by unnatural causes such as accidents and injuries.^22^, ^23^

So far, most studies have focused on predominantly male samples, in particular young adult males with ADHD in relation to traffic accidents. Here, we identified important sex differences across the lifespan. We showed that, in general men and women show opposite trends in incident occurrence, with females at lower risk in young adulthood than males but surpassing them in middle adulthood such that they have a substantially higher risk in older adulthood. However, in individuals with ADHD, this general pattern holds for severe, but not mild incidents, which form the majority of all incidents. The lifetime plot of mild incidents risk revealed that women with ADHD are at highest risk for mild incidents not only in middle and older adulthood, but across the entire adult lifespan. Related to this is that men and women with ADHD are more similar in experiencing mild incidents than men and women without ADHD, which was particularly salient in young adult women with ADHD compared with young adult women without ADHD. Both hyperactivity and impulsivity, which is more prominent in young adult males with ADHD, and attention problems, more prominent in adult females with ADHD, may contribute to incident risk.

Further, we confirmed previous findings that ADHD‐medication significantly reduces the incident risk,^24^, ^25^, ^26^ which we were able to show for mild incidents (which form the vast majority of all incidents recorded). Given a similar estimate, this seems to hold true for severe incidents as well, but the effect was not significant because of the lower statistical power within this subgroup. Although literature points to an increased risk of accidents in individuals with ADHD and comorbid substance use,^2^, ^3^ we could not confirm this. In line with the literature reporting on a decreased or not‐increased risk of incidents in individuals with ADHD and comorbid AD,^10^ we found a risk‐decreasing effect of AD and intake of psycholeptics. The presence of AD might thus counteract risk‐taking behavior in ADHD.

A major strength of this study is that findings were based on a large, representative sample of referred patients form the general German population^11^ which yielded over 17,000 adults with ADHD and a sex and age matched comparison group, allowing us to address sex, lifespan and incident severity differences. This extends existing knowledge substantially, especially for mid‐ to older adults with ADHD, who, to our knowledge, have not been studied. In addition, we combined data from all care settings, including general practitioners, which strengthens our study, given that most administrative databases that have been used to study accidents and unintentional injuries solely included referrals to specialist care^23^, ^25^ and thus on average the more severe incidents. Moreover, we add importantly to the literature by providing knowledge beyond traffic accidents.

An important limitation, however, is that claims data are first and foremost collected for billing purposes, which is not so much a limitation with regard to accidents and unintentional injuries, but it is with regard to ADHD. We could only study individuals with a documented ADHD diagnosis who were referred to a health care facility, as opposed to assessing ADHD in the general population using measurement instruments (e.g. diagnostic interviews) regardless of whether individuals with ADHD sought professional help or not.^27^ Our sample may thus be biased towards adults with more severe symptoms of ADHD, as also evident from the fact that the measured ADHD prevalence (approx. 1.5%: over 70,000 diagnosed adults with ADHD of over 4,5 million insured in the database, see Figure 1) is lower than in epidemiological studies, and potentially therefore more severe or frequent incidents. Another limitation could be that our identifying factor for ADHD was that individuals had at least one F90 diagnosis (F90.0, F90.1, F90.8, and F90.9) documented within 4 years of observation (2016–2019). Thus, we did not use F98.80 ‘Attention disorder without hyperactivity with onset in childhood and adolescence’ and as such it is possible that we studied adults with more severe symptoms of ADHD and potentially therefore more frequent incidents. However, in practice, we deem this potential bias to be very small since F98.80 is rarely used in Germany and in the rare situation that this would have occurred it is likely that a F90 was also present during the identification period of 4 years. Another limitation is that we did not stratify according to whether the presence of comorbid‐conditions and the prescription of ADHD‐medication occurred before or after incident occurrences and therefore we may have underestimated the observed protective effect of ADHD‐medication, AD and intake of psycholeptics to the extent that it was diagnosed or prescribed for the first time after the incident took place. That is, if AD, or intake ADHD‐medication or psycholeptics, occurred after an incident took place it could not have protected against the incident. Lastly, this study was restricted to a one‐year observation and incidents which may have occurred before and after were not counted. This may have led to an underestimation of the incident risk associated with ADHD.

To conclude, this study showed that individuals with ADHD have more mild and severe incidents than individuals without ADHD throughout the adult lifespan. For severe incidents, individuals with and without ADHD display a similar lifespan pattern, which only differs by the higher risk associated with ADHD: higher in young adulthood than in middle adulthood but highest in old age. For mild incidents, individuals with and without ADHD have different patterns of risk. That is, women with ADHD have a higher incident occurrence than men with ADHD throughout the adult lifespan, whereas women without ADHD only surpass men without ADHD in risk of incidents in middle adulthood and older age. Co‐occurring AD, intake of psycholeptics and ADHD‐medication buffer against the ADHD‐related increased risk. Our findings support recommendations to adequately diagnose and treat adult ADHD in order to reduce accidents and unintentional injuries.

## AUTHOR CONTRIBUTIONS

All authors meet the ICMJE criteria for authorship. Berit Libutzki, Benno Neukirch, Andreas Reif, and Catharina A. Hartman formulated the research question. Berit Libutzki (lead), Benno Neukirch, and Catharina A. Hartman designed the study, carried out data collection in collaboration with the database and analyzed the data. All authors contributed to writing the article and reviewed the article.

## CONFLICT OF INTEREST

Berit Libutzki is employed at Janssen‐Cilag AG, which, however, has no connection to this work. Benno Neukirch has nothing to declare. Sarah Kittel‐Schneider has received author's and speaker's honoraria from Medice Arzneimittel Pütter GmbH and Takeda. Andreas Reif has received honoraria and/or served on advisory boards from Medice, Shire/Takeda, Janssen, SAGE/Biogen, Boehringer Ingelheim and Cyclerion. Catharina A. Hartman declares honoraria as a speaker for Medice which has no connection to this work.

### PEER REVIEW

The peer review history for this article is available at https://publons.com/publon/10.1111/acps.13524.

## FUNDING STATEMENT

This work was supported by Hochschule Niederrhein ‐ University of Applied Sciences. The study was conducted by Hochschule Niederrhein ‐ University of Applied Sciences and the Institute for Applied Health Research Berlin (InGef).

## Supporting information


**Table S1.** Inclusion criteria and coding of comorbid condisions and medication intake, including ADHD‐medication
**Table S2.** Occurrence (%) of accidents and unintentional injuries across adulthood for males and females with and without ADHD
**Table S3.** Type of accident and unintentional injuries coded at index and within one year of observation stratified by age and sex in all individuals with and without ADHD
**Table S4.** Percentage of individuals with mental disorders and medication intake with and without accidents or unintentional injuries
**Table S5.** Multinomial Logistic Regression 2: extended model assessing ADHD, age, sex, mental comorbid disorders, medication intake and interaction effects
**Table S6.** Multinomial Logistic Regression: assessing the role of ADHD‐medication within the ADHD population on the likelihood of accidents and unintentional injuries

## Data Availability

To conduct this study authors had access to the aggregated, anonymized healthcare data as per pre‐defined study protocol. Because of the the sensitivity of the data and the German data protection laws (*Bundesdatenschutzgesetz*), raw data cannot be made available in the manuscript, the supplemental files, or in a public repository. To facilitate the replication of results, anonymized data used for this study are stored on a secure drive at the Institute for Applied Health Research Berlin (InGef). Access to the raw data used in this study can only be provided to external parties under the conditions a cooperation contract and can be accessed upon request, after written approval (info@ingef.de), if required.
